# Research trends in social media addiction and problematic social media use: A bibliometric analysis

**DOI:** 10.3389/fpsyt.2022.1017506

**Published:** 2022-11-10

**Authors:** Alfonso Pellegrino, Alessandro Stasi, Veera Bhatiasevi

**Affiliations:** ^1^Sasin School of Management, Chulalongkorn University, Bangkok, Thailand; ^2^Business Administration Division, Mahidol University International College, Mahidol University, Nakhon Pathom, Thailand

**Keywords:** bibliometric analysis, social media, social media addiction, problematic social media use, research trends

## Abstract

Despite their increasing ubiquity in people's lives and incredible advantages in instantly interacting with others, social media's impact on subjective well-being is a source of concern worldwide and calls for up-to-date investigations of the role social media plays in mental health. Much research has discovered how habitual social media use may lead to addiction and negatively affect adolescents' school performance, social behavior, and interpersonal relationships. The present study was conducted to review the extant literature in the domain of social media and analyze global research productivity during 2013–2022. Bibliometric analysis was conducted on 501 articles that were extracted from the Scopus database using the keywords social media addiction and problematic social media use. The data were then uploaded to VOSviewer software to analyze citations, co-citations, and keyword co-occurrences. Volume, growth trajectory, geographic distribution of the literature, influential authors, intellectual structure of the literature, and the most prolific publishing sources were analyzed. The bibliometric analysis presented in this paper shows that the US, the UK, and Turkey accounted for 47% of the publications in this field. Most of the studies used quantitative methods in analyzing data and therefore aimed at testing relationships between variables. In addition, the findings in this study show that most analysis were cross-sectional. Studies were performed on undergraduate students between the ages of 19–25 on the use of two social media platforms: Facebook and Instagram. Limitations as well as research directions for future studies are also discussed.

## Introduction

Social media generally refers to third-party internet-based platforms that mainly focus on social interactions, community-based inputs, and content sharing among its community of users and only feature content created by their users and not that licensed from third parties (1). Social networking sites such as Facebook, Instagram, and TikTok are prominent examples of social media that allow people to stay connected in an online world regardless of geographical distance or other obstacles (2, 3). Recent evidence suggests that social networking sites have become increasingly popular among adolescents following the strict policies implemented by many countries to counter the COVID-19 pandemic, including social distancing, “lockdowns,” and quarantine measures (4). In this new context, social media have become an essential part of everyday life, especially for children and adolescents (5). For them such media are a means of socialization that connect people together. Interestingly, social media are not only used for social communication and entertainment purposes but also for sharing opinions, learning new things, building business networks, and initiate collaborative projects (6).

Among the 7.91 billion people in the world as of 2022, 4.62 billion active social media users, and the average time individuals spent using the internet was 6 h 58 min per day with an average use of social media platforms of 2 h and 27 min (7). Despite their increasing ubiquity in people's lives and the incredible advantages they offer to instantly interact with people, an increasing number of studies have linked social media use to negative mental health consequences, such as suicidality, loneliness, and anxiety (8). Numerous sources have expressed widespread concern about the effects of social media on mental health. A 2011 report by the American Academy of Pediatrics (AAP) identifies a phenomenon known as Facebook depression which may be triggered “when preteens and teens spend a great deal of time on social media sites, such as Facebook, and then begin to exhibit classic symptoms of depression” (9). Similarly, the UK's Royal Society for Public Health (RSPH) claims that there is a clear evidence of the relationship between social media use and mental health issues based on a survey of nearly 1,500 people between the ages of 14–24 (10). According to some authors, the increase in usage frequency of social media significantly increases the risks of clinical disorders described (and diagnosed) as “Facebook depression,” “fear of missing out” (FOMO), and “social comparison orientation” (SCO) (11). Other risks include sexting (12), social media stalking (13), cyber-bullying (14), privacy breaches (15), and improper use of technology. Therefore, social media's impact on subjective well-being is a source of concern worldwide and calls for up-to-date investigations of the role social media plays with regard to mental health (8). Many studies have found that habitual social media use may lead to addiction and thus negatively affect adolescents' school performance, social behavior, and interpersonal relationships (16–18). As a result of addiction, the user becomes highly engaged with online activities motivated by an uncontrollable desire to browse through social media pages and “devoting so much time and effort to it that it impairs other important life areas” (19).

Given these considerations, the present study was conducted to review the extant literature in the domain of social media and analyze global research productivity during 2013–2022. The study presents a bibliometric overview of the leading trends with particular regard to “social media addiction” and “problematic social media use.” This is valuable as it allows for a comprehensive overview of the current state of this field of research, as well as identifies any patterns or trends that may be present. Additionally, it provides information on the geographical distribution and prolific authors in this area, which may help to inform future research endeavors.

In terms of bibliometric analysis of social media addiction research, few studies have attempted to review the existing literature in the domain extensively. Most previous bibliometric studies on social media addiction and problematic use have focused mainly on one type of screen time activity such as digital gaming or texting (20) and have been conducted with a focus on a single platform such as Facebook, Instagram, or Snapchat (21, 22). The present study adopts a more comprehensive approach by including all social media platforms and all types of screen time activities in its analysis.

Additionally, this review aims to highlight the major themes around which the research has evolved to date and draws some guidance for future research directions. In order to meet these objectives, this work is oriented toward answering the following research questions:

(1) What is the current status of research focusing on social media addiction?
(2) What are the key thematic areas in social media addiction and problematic use research?
(3) What is the intellectual structure of social media addiction as represented in the academic literature?
(4) What are the key findings of social media addiction and problematic social media research?
(5) What possible future research gaps can be identified in the field of social media addiction?

These research questions will be answered using bibliometric analysis of the literature on social media addiction and problematic use. This will allow for an overview of the research that has been conducted in this area, including information on the most influential authors, journals, countries of publication, and subject areas of study. Part 2 of the study will provide an examination of the intellectual structure of the extant literature in social media addiction while Part 3 will discuss the research methodology of the paper. Part 4 will discuss the findings of the study followed by a discussion under Part 5 of the paper. Finally, in Part 7, gaps in current knowledge about this field of research will be identified.

## Literature review

### Social media addiction research context

Previous studies on behavioral addictions have looked at a lot of different factors that affect social media addiction focusing on personality traits. Although there is some inconsistency in the literature, numerous studies have focused on three main personality traits that may be associated with social media addiction, namely anxiety, depression, and extraversion (23, 24).

It has been found that extraversion scores are strongly associated with increased use of social media and addiction to it (25, 26). People with social anxiety as well as people who have psychiatric disorders often find online interactions extremely appealing (27). The available literature also reveals that the use of social media is positively associated with being female, single, and having attention deficit hyperactivity disorder (ADHD), obsessive compulsive disorder (OCD), or anxiety (28).

In a study by Seidman (29), the Big Five personality traits were assessed using Saucier's (30) Mini-Markers Scale. Results indicated that neurotic individuals use social media as a safe place for expressing their personality and meet belongingness needs. People affected by neurosis tend to use online social media to stay in touch with other people and feel better about their social lives (31). Narcissism is another factor that has been examined extensively when it comes to social media, and it has been found that people who are narcissistic are more likely to become addicted to social media (32). In this case users want to be seen and get “likes” from lots of other users. Longstreet and Brooks (33) did a study on how life satisfaction depends on how much money people make. Life satisfaction was found to be negatively linked to social media addiction, according to the results. When social media addiction decreases, the level of life satisfaction rises. But results show that in lieu of true-life satisfaction people use social media as a substitute (for temporary pleasure vs. longer term happiness).

Researchers have discovered similar patterns in students who tend to rank high in shyness: they find it easier to express themselves online rather than in person (34, 35). With the use of social media, shy individuals have the opportunity to foster better quality relationships since many of their anxiety-related concerns (e.g., social avoidance and fear of social devaluation) are significantly reduced (36, 37).

### Problematic use of social media

The amount of research on problematic use of social media has dramatically increased since the last decade. But using social media in an unhealthy manner may not be considered an addiction or a disorder as this behavior has not yet been formally categorized as such (38). Although research has shown that people who use social media in a negative way often report negative health-related conditions, most of the data that have led to such results and conclusions comprise self-reported data (39). The dimensions of excessive social media usage are not exactly known because there are not enough diagnostic criteria and not enough high-quality long-term studies available yet. This is what Zendle and Bowden-Jones (40) noted in their own research. And this is why terms like “problematic social media use” have been used to describe people who use social media in a negative way. Furthermore, if a lot of time is spent on social media, it can be hard to figure out just when it is being used in a harmful way. For instance, people easily compare their appearance to what they see on social media, and this might lead to low self-esteem if they feel they do not look as good as the people they are following. According to research in this domain, the extent to which an individual engages in photo-related activities (e.g., taking selfies, editing photos, checking other people's photos) on social media is associated with negative body image concerns. Through curated online images of peers, adolescents face challenges to their self-esteem and sense of self-worth and are increasingly isolated from face-to-face interaction.

To address this problem the Diagnostic and Statistical Manual of Mental Disorders (DSM-V) has been used by some scholars (41, 42). These scholars have used criteria from the DSM-V to describe one problematic social media use, internet gaming disorder, but such criteria could also be used to describe other types of social media disorders. Franchina et al. (43) and Scott and Woods (44), for example, focus their attention on individual-level factors (like fear of missing out) and family-level factors (like childhood abuse) that have been used to explain why people use social media in a harmful way. Friends-level factors have also been explored as a social well-being measurement to explain why people use social media in a malevolent way and demonstrated significant positive correlations with lower levels of friend support (45). Macro-level factors have also been suggested, such as the normalization of surveillance (46) and the ability to see what people are doing online (47). Gender and age seem to be highly associated to the ways people use social media negatively. Particularly among girls, social media use is consistently associated with mental health issues (41, 48, 49), an association more common among older girls than younger girls (46, 48).

Most studies have looked at the connection between social media use and its effects (such as social media addiction) and a number of different psychosomatic disorders. In a recent study conducted by Vannucci and Ohannessian (50), the use of social media appears to have a variety of effects “on psychosocial adjustment during early adolescence, with high social media use being the most problematic.” It has been found that people who use social media in a harmful way are more likely to be depressed, anxious, have low self-esteem, be more socially isolated, have poorer sleep quality, and have more body image dissatisfaction. Furthermore, harmful social media use has been associated with unhealthy lifestyle patterns (for example, not getting enough exercise or having trouble managing daily obligations) as well as life threatening behaviors such as illicit drug use, excessive alcohol consumption and unsafe sexual practices (51, 52).

A growing body of research investigating social media use has revealed that the extensive use of social media platforms is correlated with a reduced performance on cognitive tasks and in mental effort (53). Overall, it appears that individuals who have a problematic relationship with social media or those who use social media more frequently are more likely to develop negative health conditions.

### Social media addiction and problematic use systematic reviews

Previous studies have revealed the detrimental impacts of social media addiction on users' health. A systematic review by Khan and Khan (20) has pointed out that social media addiction has a negative impact on users' mental health. For example, social media addiction can lead to stress levels rise, loneliness, and sadness (54). Anxiety is another common mental health problem associated with social media addiction. Studies have found that young adolescents who are addicted to social media are more likely to suffer from anxiety than people who are not addicted to social media (55). In addition, social media addiction can also lead to physical health problems, such as obesity and carpal tunnel syndrome a result of spending too much time on the computer (22).

Apart from the negative impacts of social media addiction on users' mental and physical health, social media addiction can also lead to other problems. For example, social media addiction can lead to financial problems. A study by Sharif and Yeoh (56) has found that people who are addicted to social media tend to spend more money than those who are not addicted to social media. In addition, social media addiction can also lead to a decline in academic performance. Students who are addicted to social media are more likely to have lower grades than those who are not addicted to social media (57).

## Research methodology

### Bibliometric analysis

Merigo et al. (58) use bibliometric analysis to examine, organize, and analyze a large body of literature from a quantitative, objective perspective in order to assess patterns of research and emerging trends in a certain field. A bibliometric methodology is used to identify the current state of the academic literature, advance research. and find objective information (59). This technique allows the researchers to examine previous scientific work, comprehend advancements in prior knowledge, and identify future study opportunities.

To achieve this objective and identify the research trends in social media addiction and problematic social media use, this study employs two bibliometric methodologies: performance analysis and science mapping. Performance analysis uses a series of bibliometric indicators (e.g., number of annual publications, document type, source type, journal impact factor, languages, subject area, h-index, and countries) and aims at evaluating groups of scientific actors on a particular topic of research. VOSviewer software (60) was used to carry out the science mapping. The software is used to visualize a particular body of literature and map the bibliographic material using the co-occurrence analysis of author, index keywords, nations, and fields of publication (61, 62).

### Data collection

**Figure 1:**
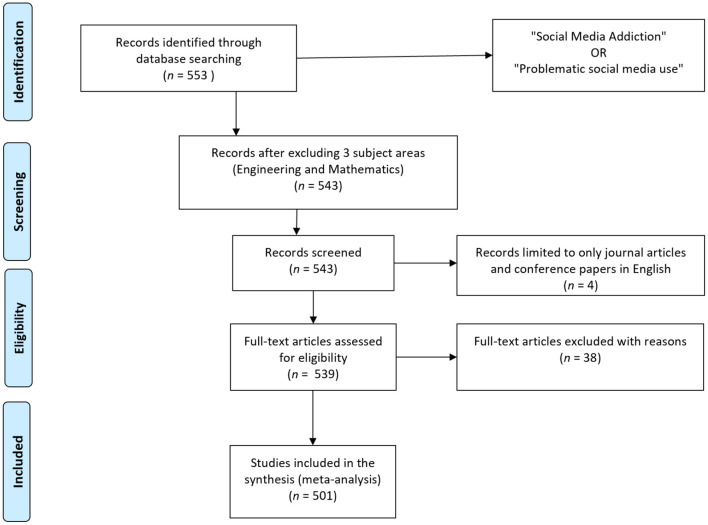
Preferred reporting items for systematic reviews and meta-analysis (PRISMA) flowchart showing the search procedures used in the review.

After picking keywords, designing the search strings, and building up a database, the authors conducted a bibliometric literature search. Scopus was utilized to gather exploration data since it is a widely used database that contains the most comprehensive view of the world's research output and provides one of the most effective search engines. If the research was to be performed using other database such as Web Of Science or Google Scholar the authors may have obtained larger number of articles however they may not have been all particularly relevant as Scopus is known to have the most widest and most relevant scholar search engine in marketing and social science. A keyword search for “social media addiction” OR “problematic social media use” yielded 553 papers, which were downloaded from Scopus. The information was gathered in March 2022, and because the Scopus database is updated on a regular basis, the results may change in the future. Next, the authors examined the titles and abstracts to see whether they were relevant to the topics treated. There were two common grounds for document exclusion. First, while several documents emphasized the negative effects of addiction in relation to the internet and digital media, they did not focus on social networking sites specifically. Similarly, addiction and problematic consumption habits were discussed in relation to social media in several studies, although only in broad terms. This left a total of 511 documents. Articles were then limited only to journal articles, conference papers, reviews, books, and only those published in English. This process excluded 10 additional documents. Then, the relevance of the remaining articles was finally checked by reading the titles, abstracts, and keywords. Documents were excluded if social networking sites were only mentioned as a background topic or very generally. This resulted in a final selection of 501 research papers, which were then subjected to bibliometric analysis (see Figure 1).

After identifying 501 Scopus files, bibliographic data related to these documents were imported into an Excel sheet where the authors' names, their affiliations, document titles, keywords, abstracts, and citation figures were analyzed. These were subsequently uploaded into VOSViewer software version 1.6.8 to begin the bibliometric review. Descriptive statistics were created to define the whole body of knowledge about social media addiction and problematic social media use. VOSViewer was used to analyze citation, co-citation, and keyword co-occurrences. According to Zupic and Cater (63), co-citation analysis measures the influence of documents, authors, and journals heavily cited and thus considered influential. Co-citation analysis has the objective of building similarities between authors, journals, and documents and is generally defined as the frequency with which two units are cited together within the reference list of a third article.

The implementation of social media addiction performance analysis was conducted according to the models recently introduced by Karjalainen et al. (64) and Pattnaik (65). Throughout the manuscript there are operational definitions of relevant terms and indicators following a standardized bibliometric approach. The cumulative academic impact (CAI) of the documents was measured by the number of times they have been cited in other scholarly works while the fine-grained academic impact (FIA) was computed according to the authors citation analysis and authors co-citation analysis within the reference lists of documents that have been specifically focused on social media addiction and problematic social media use.

## Results

Results of the study presented here include the findings on social media addiction and social media problematic use. The results are presented by the foci outlined in the study questions.

### Volume, growth trajectory, and geographic distribution of the literature

**Figure 2:**
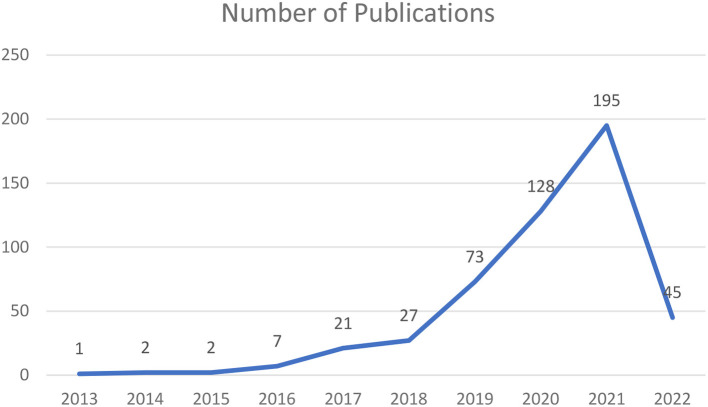
Annual volume of social media addiction or social media problematic use (*n* = 501).

After performing the Scopus-based investigation of the current literature regarding social media addiction and problematic use of social media, the authors obtained a knowledge base consisting of 501 documents comprising 455 journal articles, 27 conference papers, 15 articles reviews, 3 books and 1 conference review. The included literature was very recent. As shown in Figure 2, publication rates started very slowly in 2013 but really took off in 2018, after which publications dramatically increased each year until a peak was reached in 2021 with 195 publications. Analyzing the literature published during the past decade reveals an exponential increase in scholarly production on social addiction and its problematic use. This might be due to the increasingly widespread introduction of social media sites in everyday life and the ubiquitous diffusion of mobile devices that have fundamentally impacted human behavior. The dip in the number of publications in 2022 is explained by the fact that by the time the review was carried out the year was not finished yet and therefore there are many articles still in press.

**Figure 3:**
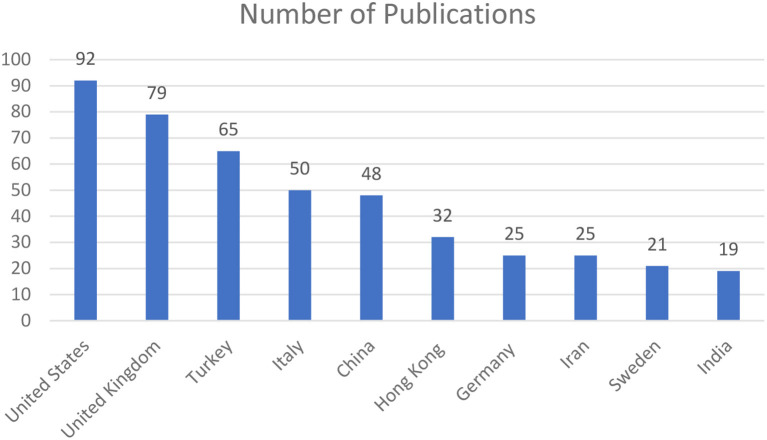
Global dispersion of social networking sites in relation to social media addiction or social media problematic use.

The geographical distribution trends of scholarly publications on social media addiction or problematic use of social media are highlighted in Figure 3. The articles were assigned to a certain country according to the nationality of the university with whom the first author was affiliated with. The figure shows that the most productive countries are the USA (92), the U.K. (79), and Turkey (63), which combined produced 236 articles, equal to 47% of the entire scholarly production examined in this bibliometric analysis. Turkey has slowly evolved in various ways with the growth of the internet and social media. Anglo-American scholarly publications on problematic social media consumer behavior represent the largest research output. Yet it is interesting to observe that social networking sites studies are attracting many researchers in Asian countries, particularly China. For many Chinese people, social networking sites are a valuable opportunity to involve people in political activism in addition to simply making purchases (66).

### Analysis of influential authors

This section analyses the high-impact authors in the Scopus-indexed knowledge base on social networking sites in relation to social media addiction or problematic use of social media. It provides valuable insights for establishing patterns of knowledge generation and dissemination of literature about social networking sites relating to addiction and problematic use.

Table 1 acknowledges the top 10 most highly cited authors with the highest total citations in the database.

**Table 1:** Highly cited authors on social media addiction and problematic use (*n* = 501).

**Rank**	**Author**	**Country**	**Articles**	**Citations**	**Total link^a^ strength**
1	Griffiths, MD	UK	65	2175	367
2	Lin, CY	Taiwan	20	576	278
3	Pakpour, AH	Sweden	18	553	260
4	Demetrovics, Z	Hungary	12	475	82
5	Chen, IH	Hong Kong	10	255	162
6	Kircaburun, K	Turkey	10	252	31
7	Kuss, DJ	UK	13	199	20
8	Turel, O	USA	10	190	18
9	Casale, S	Italy	9	115	22
10	Montag, C	Germany	10	79	12

^a^Total link strength indicates the number of publications in which an author occurs.

Table 1 shows that MD Griffiths (sixty-five articles), CY Lin (twenty articles), and AH Pakpour (eighteen articles) are the most productive scholars according to the number of Scopus documents examined in the area of *social media addiction and its problematic use*. If the criteria are changed and authors ranked according to the overall number of citations received in order to determine high-impact authors, the same three authors turn out to be the most highly cited authors. It should be noted that these highly cited authors tend to enlist several disciplines in examining social media addiction and problematic use. Griffiths, for example, focuses on behavioral addiction stemming from not only digital media usage but also from gambling and video games. Lin, on the other hand, focuses on the negative effects that the internet and digital media can have on users' mental health, and Pakpour approaches the issue from a behavioral medicine perspective.

### Intellectual structure of the literature

**Figure 4:**
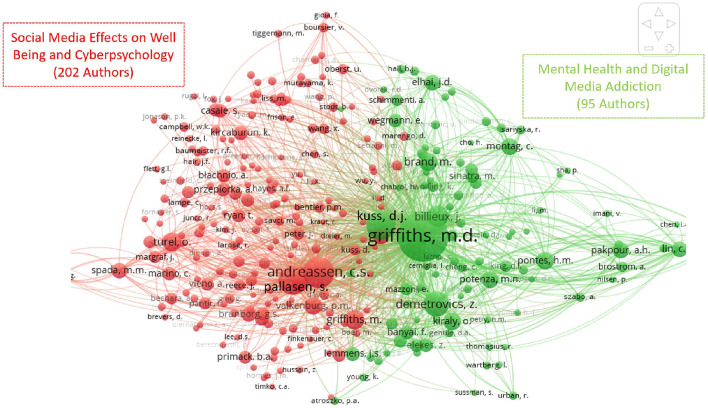
Two clusters, representing the intellectual structure of the social media and its problematic use literature.

In this part of the paper, the authors illustrate the “intellectual structure” of the social media addiction and the problematic use of social media's literature. An author co-citation analysis (ACA) was performed which is displayed as a figure that depicts the relations between highly co-cited authors. The study of co-citation assumes that strongly co-cited authors carry some form of intellectual similarity (67). Figure 4 shows the author co-citation map. Nodes represent units of analysis (in this case scholars) and network ties represent similarity connections. Nodes are sized according to the number of co-citations received—the bigger the node, the more co-citations it has. Adjacent nodes are considered intellectually similar.

Scholars belonging to the green cluster (Mental Health and Digital Media Addiction) have extensively published on medical analysis tools and how these can be used to heal users suffering from addiction to digital media, which can range from gambling, to internet, to videogame addictions. Scholars in this school of thought focus on the negative effects on users' mental health, such as depression, anxiety, and personality disturbances. Such studies focus also on the role of screen use in the development of mental health problems and the increasing use of medical treatments to address addiction to digital media. They argue that addiction to digital media should be considered a mental health disorder and treatment options should be made available to users.

In contrast, scholars within the red cluster (Social Media Effects on Well Being and Cyberpsychology) have focused their attention on the effects of social media toward users' well-being and how social media change users' behavior, focusing particular attention on the human-machine interaction and how methods and models can help protect users' well-being. Two hundred and two authors belong to this group, the top co-cited being Andreassen (667 co-citations), Pallasen (555 co-citations), and Valkenburg (215 co-citations). These authors have extensively studied the development of addiction to social media, problem gambling, and internet addiction. They have also focused on the measurement of addiction to social media, cyberbullying, and the dark side of social media.

### Most influential source title in the field of social media addiction and its problematic use

To find the preferred periodicals in the field of social media addiction and its problematic use, the authors have selected 501 articles published in 263 journals. Table 2 gives a ranked list of the top 10 journals that constitute the core publishing sources in the field of social media addiction research. In doing so, the authors analyzed the journal's impact factor, Scopus Cite Score, h-index, quartile ranking, and number of publications per year.

**Table 2:** Top 10 most cited and more frequently mentioned documents in the field of social media addiction.

**Rank**	**Title**	**Authors**	**Journal**	**Year**	**Total citations**	**Total citation per year**
1	The relationship between addictive use of social media, narcissism, and self-esteem: Findings from a large national survey	Andreassen et al.	Addictive behaviors	2017	405	81
2	Problematic social media use: Results from a large-scale nationally representative adolescent sample	Bányai et al.	PloS one	2017	328	65.5
3	Extraversion, neuroticism, attachment style and fear of missing out as predictors of social media use and addiction	Blackwell et al.	Personality and Individual Differences	2017	298	59.6
4	The social media disorder scale	Van Den Eijnden et al.	Computers in Human Behavior	2016	279	46.5
5	Association between social media use and depression among US young adults	Lin et al.	Depression and anxiety	2016	274	45.7
6	The relations among social media addiction, self-esteem, and life satisfaction in university students	Hawi and Samaha	Social Science Computer Review	2017	149	29.8
7	Social networking addiction, attachment style, and validation of the Italian version of the Bergen Social Media Addiction Scale	Monacis et al.	Journal of Behavioral Addictions	2017	132	26.4
8	Determinants of phubbing, which is the sum of many virtual addictions: A structural equation model	Karadag et al.	Journal of Behavioral Addictions	2015	128	18.2
9	Investigating mediated effects of fear of COVID-19 and COVID-19 misunderstanding in the association between problematic social media use, psychological distress, and insomnia	Lin et al.	Internet interventions	2020	97	48.5
10	Relationships between severity of internet gaming disorder, severity of problematic social media use, sleep quality and psychological distress	Wong et al.	International Journal of Environmental Research and Public Health	2020	95	47.5

The journal *Addictive Behaviors* topped the list, with 700 citations and 22 publications (4.3%), followed by *Computers in Human Behaviors*, with 577 citations and 13 publications (2.5%), *Journal of Behavioral Addictions*, with 562 citations and 17 publications (3.3%), and *International Journal of Mental Health and Addiction*, with 502 citations and 26 publications (5.1%). Five of the 10 most productive journals in the field of social media addiction research are published by Elsevier (all Q1 rankings) while Springer and Frontiers Media published one journal each.

Documents citation analysis identified the most influential and most frequently mentioned documents in a certain scientific field. Andreassen has received the most citations among the 10 most significant papers on social media addiction, with 405 (Table 2). The main objective of this type of studies was to identify the associations and the roles of different variables as predictors of social media addiction (e.g., (19, 68, 69)). According to general addiction models, the excessive and problematic use of digital technologies is described as “being overly concerned about social media, driven by an uncontrollable motivation to log on to or use social media, and devoting so much time and effort to social media that it impairs other important life areas” (27, 70). Furthermore, the purpose of several highly cited studies (31, 71) was to analyse the connections between young adults' sleep quality and psychological discomfort, depression, self-esteem, and life satisfaction and the severity of internet and problematic social media use, since the health of younger generations and teenagers is of great interest this may help explain the popularity of such papers. Despite being the most recent publication Lin et al.'s work garnered more citations annually. The desire to quantify social media addiction in individuals can also help explain the popularity of studies which try to develop measurement scales (42, 72). Some of the highest-ranked publications are devoted to either the presentation of case studies or testing relationships among psychological constructs (73).

### Keyword co-occurrence analysis

The research question, “What are the key thematic areas in social media addiction literature?” was answered using keyword co-occurrence analysis. Keyword co-occurrence analysis is conducted to identify research themes and discover keywords. It mainly examines the relationships between co-occurrence keywords in a wide variety of literature (74). In this approach, the idea is to explore the frequency of specific keywords being mentioned together.

**Figure 5:**
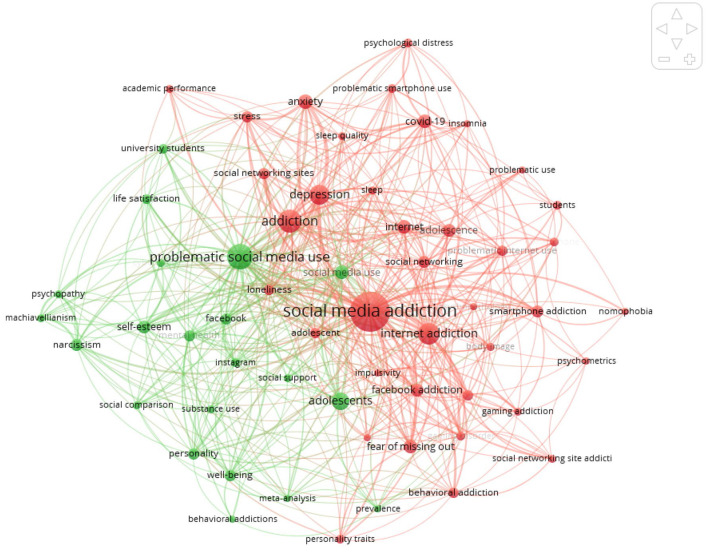
Keywords co-occurrence map. Threshold: 5 co-occurrences.

Utilizing VOSviewer, the authors conducted a keyword co-occurrence analysis to characterize and review the developing trends in the field of social media addiction. The top 10 most frequent keywords are presented in Table 3. The results indicate that “social media addiction” is the most frequent keyword (178 occurrences), followed by “problematic social media use” (74 occurrences), “internet addiction” (51 occurrences), and “depression” (46 occurrences). As shown in the co-occurrence network (Figure 5), the keywords can be grouped into two major clusters. “Problematic social media use” can be identified as the core theme of the green cluster. In the red cluster, keywords mainly identify a specific aspect of problematic social media use: social media addiction.

**Table 3:** Frequency of occurrence of top 10 keywords.

**Keyword**	**Occurrences**
Social media addiction	178
Problematic social media use	74
Internet addiction	51
Depression	46
Adolescents	35
Anxiety	25
COVID-19	22
Internet	22
Social media use	22
Self-esteem	20

The results of the keyword co-occurrence analysis for journal articles provide valuable perspectives and tools for understanding concepts discussed in past studies of social media usage (75). More precisely, it can be noted that there has been a large body of research on social media addiction together with other types of technological addictions, such as compulsive web surfing, internet gaming disorder, video game addiction and compulsive online shopping (76–78). This field of research has mainly been directed toward teenagers, middle school students, and college students and university students in order to understand the relationship between social media addiction and mental health issues such as depression, disruptions in self-perceptions, impairment of social and emotional activity, anxiety, neuroticism, and stress (79–81).

## Discussion

The findings presented in this paper show that there has been an exponential increase in scholarly publications—from two publications in 2013 to 195 publications in 2021. There were 45 publications in 2022 at the time this study was conducted. It was interesting to observe that the US, the UK, and Turkey accounted for 47% of the publications in this field even though none of these countries are in the top 15 countries in terms of active social media penetration (82) although the US has the third highest number of social media users (83). Even though China and India have the highest number of social media users (83), first and second respectively, they rank fifth and tenth in terms of publications on social media addiction or problematic use of social media. In fact, the US has almost double the number of publications in this field compared to China and almost five times compared to India. Even though East Asia, Southeast Asia, and South Asia make up the top three regions in terms of worldwide social media users (84), except for China and India there have been only a limited number of publications on social media addiction or problematic use. An explanation for that could be that there is still a lack of awareness on the negative consequences of the use of social media and the impact it has on the mental well-being of users. More research in these regions should perhaps be conducted in order to understand the problematic use and addiction of social media so preventive measures can be undertaken.

From the bibliometric analysis, it was found that most of the studies examined used quantitative methods in analyzing data and therefore aimed at testing relationships between variables. In addition, many studies were empirical, aimed at testing relationships based on direct or indirect observations of social media use. Very few studies used theories and for the most part if they did they used the technology acceptance model and social comparison theories. The findings presented in this paper show that none of the studies attempted to create or test new theories in this field, perhaps due to the lack of maturity of the literature. Moreover, neither have very many qualitative studies been conducted in this field. More qualitative research in this field should perhaps be conducted as it could explore the motivations and rationales from which certain users' behavior may arise.

The authors found that almost all the publications on social media addiction or problematic use relied on samples of undergraduate students between the ages of 19–25. The average daily time spent by users worldwide on social media applications was highest for users between the ages of 40–44, at 59.85 min per day, followed by those between the ages of 35–39, at 59.28 min per day, and those between the ages of 45–49, at 59.23 per day (85). Therefore, more studies should be conducted exploring different age groups, as users between the ages of 19–25 do not represent the entire population of social media users. Conducting studies on different age groups may yield interesting and valuable insights to the field of social media addiction. For example, it would be interesting to measure the impacts of social media use among older users aged 50 years or older who spend almost the same amount of time on social media as other groups of users (56.43 min per day) (85).

A majority of the studies tested social media addiction or problematic use based on only two social media platforms: Facebook and Instagram. Although Facebook and Instagram are ranked first and fourth in terms of most popular social networks by number of monthly users, it would be interesting to study other platforms such as YouTube, which is ranked second, and WhatsApp, which is ranked third (86). Furthermore, TikTok would also be an interesting platform to study as it has grown in popularity in recent years, evident from it being the most downloaded application in 2021, with 656 million downloads (87), and is ranked second in Q1 of 2022 (88). Moreover, most of the studies focused only on one social media platform. Comparing different social media platforms would yield interesting results because each platform is different in terms of features, algorithms, as well as recommendation engines. The purpose as well as the user behavior for using each platform is also different, therefore why users are addicted to these platforms could provide a meaningful insight into social media addiction and problematic social media use.

Lastly, most studies were cross-sectional, and not longitudinal, aiming at describing results over a certain point in time and not over a long period of time. A longitudinal study could better describe the long-term effects of social media use.

## Conclusion

This study was conducted to review the extant literature in the field of social media and analyze the global research productivity during the period ranging from 2013 to 2022. The study presents a bibliometric overview of the leading trends with particular regard to “social media addiction” and “problematic social media use.” The authors applied science mapping to lay out a knowledge base on social media addiction and its problematic use. This represents the first large-scale analysis in this area of study.

A keyword search of “social media addiction” OR “problematic social media use” yielded 553 papers, which were downloaded from Scopus. After performing the Scopus-based investigation of the current literature regarding social media addiction and problematic use, the authors ended up with a knowledge base consisting of 501 documents comprising 455 journal articles, 27 conference papers, 15 articles reviews, 3 books, and 1 conference review.

The geographical distribution trends of scholarly publications on social media addiction or problematic use indicate that the most productive countries were the USA (92), the U.K. (79), and Turkey (63), which together produced 236 articles. Griffiths (sixty-five articles), Lin (twenty articles), and Pakpour (eighteen articles) were the most productive scholars according to the number of Scopus documents examined in the area of social media addiction and its problematic use. An author co-citation analysis (ACA) was conducted which generated a layout of social media effects on well-being and cyber psychology as well as mental health and digital media addiction in the form of two research literature clusters representing the intellectual structure of social media and its problematic use.

The preferred periodicals in the field of social media addiction and its problematic use were *Addictive Behaviors*, with 700 citations and 22 publications, followed by *Computers in Human Behavior*, with 577 citations and 13 publications, and *Journal of Behavioral Addictions*, with 562 citations and 17 publications. Keyword co-occurrence analysis was used to investigate the key thematic areas in the social media literature, as represented by the top three keyword phrases in terms of their frequency of occurrence, namely, “social media addiction,” “problematic social media use,” and “social media addiction.”

This research has a few limitations. The authors used science mapping to improve the comprehension of the literature base in this review. First and foremost, the authors want to emphasize that science mapping should not be utilized in place of established review procedures, but rather as a supplement. As a result, this review can be considered the initial stage, followed by substantive research syntheses that examine findings from recent research. Another constraint stems from how 'social media addiction' is defined. The authors overcame this limitation by inserting the phrase “social media addiction” OR “problematic social media use” in the search string. The exclusive focus on SCOPUS-indexed papers creates a third constraint. The SCOPUS database has a larger number of papers than does Web of Science although it does not contain all the publications in a given field.

Although the total body of literature on social media addiction is larger than what is covered in this review, the use of co-citation analyses helped to mitigate this limitation. This form of bibliometric study looks at all the publications listed in the reference list of the extracted SCOPUS database documents. As a result, a far larger dataset than the one extracted from SCOPUS initially has been analyzed.

The interpretation of co-citation maps should be mentioned as a last constraint. The reason is that the procedure is not always clear, so scholars must have a thorough comprehension of the knowledge base in order to make sense of the result of the analysis (63). This issue was addressed by the authors' expertise, but it remains somewhat subjective.

## Implications

The findings of this study have implications mainly for government entities and parents. The need for regulation of social media addiction is evident when considering the various risks associated with habitual social media use. Social media addiction may lead to negative consequences for adolescents' school performance, social behavior, and interpersonal relationships. In addition, social media addiction may also lead to other risks such as sexting, social media stalking, cyber-bullying, privacy breaches, and improper use of technology. Given the seriousness of these risks, it is important to have regulations in place to protect adolescents from the harms of social media addiction.

### Regulation of social media platforms

One way that regulation could help protect adolescents from the harms of social media addiction is by limiting their access to certain websites or platforms. For example, governments could restrict adolescents' access to certain websites or platforms during specific hours of the day. This would help ensure that they are not spending too much time on social media and are instead focusing on their schoolwork or other important activities.

Another way that regulation could help protect adolescents from the harms of social media addiction is by requiring companies to put warning labels on their websites or apps. These labels would warn adolescents about the potential risks associated with excessive use of social media.

Finally, regulation could also require companies to provide information about how much time each day is recommended for using their website or app. This would help adolescents make informed decisions about how much time they want to spend on social media each day. These proposed regulations would help to protect children from the dangers of social media, while also ensuring that social media companies are more transparent and accountable to their users.

### Parental involvement in adolescents' social media use

Parents should be involved in their children's social media use to ensure that they are using these platforms safely and responsibly. Parents can monitor their children's online activity, set time limits for social media use, and talk to their children about the risks associated with social media addiction.

### Education on responsible social media use

Adolescents need to be educated about responsible social media use so that they can enjoy the benefits of these platforms while avoiding the risks associated with addiction. Education on responsible social media use could include topics such as cyber-bullying, sexting, and privacy breaches.

## Research directions for future studies

A content analysis was conducted to answer the fifth research questions “What are the potential research directions for addressing social media addiction in the future?” The study reveals that there is a lack of screening instruments and diagnostic criteria to assess social media addiction. Validated DSM-V-based instruments could shed light on the factors behind social media use disorder. Diagnostic research may be useful in order to understand social media behavioral addiction and gain deeper insights into the factors responsible for psychological stress and psychiatric disorders. In addition to cross-sectional studies, researchers should also conduct longitudinal studies and experiments to assess changes in users' behavior over time (20).

Another important area to examine is the role of engagement-based ranking and recommendation algorithms in online habit formation. More research is required to ascertain how algorithms determine which content type generates higher user engagement. A clear understanding of the way social media platforms gather content from users and amplify their preferences would lead to the development of a standardized conceptualization of social media usage patterns (89). This may provide a clearer picture of the factors that lead to problematic social media use and addiction. It has been noted that “misinformation, toxicity, and violent content are inordinately prevalent” in material reshared by users and promoted by social media algorithms (90).

Additionally, an understanding of engagement-based ranking models and recommendation algorithms is essential in order to implement appropriate public policy measures. To address the specific behavioral concerns created by social media, legislatures must craft appropriate statutes. Thus, future qualitative research to assess engagement based ranking frameworks is extremely necessary in order to provide a broader perspective on social media use and tackle key regulatory gaps. Particular emphasis must be placed on consumer awareness, algorithm bias, privacy issues, ethical platform design, and extraction and monetization of personal data (91).

From a geographical perspective, the authors have identified some main gaps in the existing knowledge base that uncover the need for further research in certain regions of the world. Accordingly, the authors suggest encouraging more studies on internet and social media addiction in underrepresented regions with high social media penetration rates such as Southeast Asia and South America. In order to draw more contributions from these countries, journals with high impact factors could also make specific calls. This would contribute to educating social media users about platform usage and implement policy changes that support the development of healthy social media practices.

The authors hope that the findings gathered here will serve to fuel interest in this topic and encourage other scholars to investigate social media addiction in other contexts on newer platforms and among wide ranges of sample populations. In light of the rising numbers of people experiencing mental health problems (e.g., depression, anxiety, food disorders, and substance addiction) in recent years, it is likely that the number of papers related to social media addiction and the range of countries covered will rise even further.

## Data availability statement

The original contributions presented in the study are included in the article/supplementary material, further inquiries can be directed to the corresponding authors.

## Author contributions

AP took care of bibliometric analysis and drafting the paper. VB took care of proofreading and adding value to the paper. AS took care of the interpretation of the findings. All authors contributed to the article and approved the submitted version.

## Conflict of interest

The authors declare that the research was conducted in the absence of any commercial or financial relationships that could be construed as a potential conflict of interest.

## Publisher's note

All claims expressed in this article are solely those of the authors and do not necessarily represent those of their affiliated organizations, or those of the publisher, the editors and the reviewers. Any product that may be evaluated in this article, or claim that may be made by its manufacturer, is not guaranteed or endorsed by the publisher.
